# Learning to care: medical students’ reported value and evaluation of palliative care teaching involving meeting patients and reflective writing

**DOI:** 10.1186/s12909-016-0827-6

**Published:** 2016-11-25

**Authors:** Erica Borgstrom, Rachel Morris, Diana Wood, Simon Cohn, Stephen Barclay

**Affiliations:** 1 Faculty of Wellbeing, Education and Language Studies, Open University, Milton Keynes, MK7 6AA UK; 2 Primary Care Unit, Department of Public Health and Primary Care, Institute of Public Health, University of Cambridge, Cambridge, CB2 0SR UK; 3 University of Cambridge School of Clinical Medicine, Cambridge, CB2 0SP UK; 4 Department of Health Services Research and Policy, London School of Hygiene and Tropical Medicine, London, WC1N 9SH UK

**Keywords:** Reflective practice, Medical education, Palliative care, End of life care, Reflection, Curriculum development

## Abstract

**Background:**

Over recent years there has been an increase in teaching of both palliative care and reflective practice in UK medical schools. The palliative care teaching at the University of Cambridge School of Clinical Medicine is multi-faceted and involves students writing reflective essays after individually meeting patients approaching the end of life during their final year general practice and hospital medicine placements. This paper draws on two studies examining this teaching element to analyse what the students found valuable about it and to comment on the practice of meeting patients and subsequent reflective writing.

**Methods:**

Two studies have explored students’ perceptions of these course components. The first was a thematic analysis of 234 reflective essays from 123 students written in 2007-2008, including examining what students wrote about the exercise itself. The second project involved a semi-structured questionnaire that students completed anonymously; this paper reports on the free text elements of that study [sample size =107]. Since similar themes were found in both studies, the coding structures from each project were compared and combined, enabling triangulation of the findings around what the students found valuable from the palliative care teaching involving meeting patients and reflective writing.

**Results:**

Overall, students reported that these components of the palliative care teaching are valuable. Four main themes were identified as aspects that students valued: (1) dedicated time with patients, (2) learning about wider elements of treatment and holistic care, (3) practicing communication skills, and (4) learning about themselves through reflective writing. Some students expressed a dislike for having to formally write a reflective essay.

**Conclusion:**

It is possible to arrange for all of the medical students to individually meet at least two patients receiving palliative or end of life care. Students found these encounters valuable and many wrote about the benefit of formally writing about these experiences. Students reported finding this model useful in widening their skill-set and understanding of palliative care.

**Electronic supplementary material:**

The online version of this article (doi:10.1186/s12909-016-0827-6) contains supplementary material, which is available to authorized users.

## Background

Teaching in palliative and end of life care has increased within British medical schools over the last 20 years [1, 2]. Once thought to be too difficult or inappropriate, students now routinely encounter the issues and practicalities of caring for people approaching the end of their lives, although the form and content of teaching varies considerably both nationally and internationally [3–5]. In the UK, the recently revised Association for Palliative Medicine national curriculum for medical student education recommends the main areas to be addressed, including: basic principles; physical care; psychosocial care; communication; social and family relationships; grief and bereavement; personal and professional issues; and ethical and legal issues [6]. Each medical school is developing their own way of addressing the knowledge and skills that students will need when providing palliative and end of life care [7].

At the same time as the rise in palliative care teaching, there has been an increased emphasis on reflection within medicine. Reflective practice is considered a core element of professionalism [8] and the ability to reflect on one’s own performance is now seen to be a crucial skill for personal and professional development. Reflection has therefore become an important element of the appraisal and revalidation processes [9]. Self-reflection can take place in many different forms including one-to-one mentoring, small group discussions and written methods such as diaries, portfolios and essays and is increasingly being used in undergraduate medical education [10]. Reflective writing is one way to incorporate reflection into medical teaching and has some advantages over face-to-face methods in that it is easier to administer and assess [11]. However there is evidence that reflective writing is not of itself adequate to teach reflective practice: it has not been shown to improve the quality or level of reflection [10] though it may be valuable in other ways [12]. Given the range of professional and personal issues that arise during encounters with patients approaching the end of their lives, [13] these instances provide a valuable opportunity for such reflective teaching.

Acknowledging the value of student feedback in appraising teaching methods and course content, [14, 15] this paper outlines students’ views of the value of meeting patients and writing reflective palliative care essays at the University of Cambridge School of Clinical Medicine, based on two studies conducted between 2008 and 2010. This paper outlines what the students found valuable and what some found disagreeable: it is hoped that this will inform similar curricular developments in other medical schools.

### Background to teaching

The University of Cambridge School of Clinical Medicine palliative care course includes reflective writing during the final year, arising from individual meetings with two patients approaching the end of their lives, during senior General Practice (GP) and senior Medicine placements. Prior to this final year, students have ten half day palliative care sessions covering symptom management, service provision, the needs of different patient groups, and ethical and communication issues. At the time of these studies, SB led the palliative care course component and RM led the professionalism component, which includes a focus on reflective practice; the other authors are not involved in delivering teaching on these components.

**Fig. 1:**
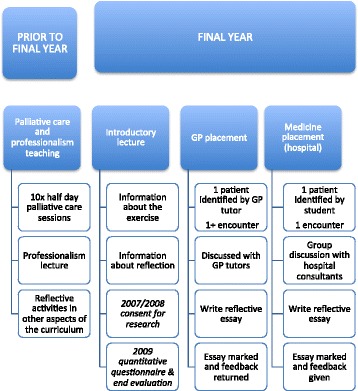
Palliative care & reflective essay teaching

The objectives of this component of the course are: to enable students to have in-depth contact with patients near the end of life, to discuss these cases with tutors, to write reflective essay based on those patient encounters, to learn more about palliative care, and to develop reflective abilities. Assessment of the essays evaluates the latter three objectives. This paper outlines how one element of palliative care teaching at the University of Cambridge School of Clinical Medicine is addressing the main areas of palliative care through the use of patient encounters and reflective essays, and what students say they value, or not, from this teaching. Figure 1 depicts the palliative care teaching element that this paper is about: using encounters with patients as the basis for reflective writing in the final year of medical school.

### Description of the teaching exercise

In the autumn of their final academic year, students have an introductory lecture about the reflective essays, outlining the logistics of identifying and meeting the patients and the expected content and structure of reflective essays. The lecture includes an introduction to Glaser and Strauss’ *Awareness of Dying* [16] and Kubler-Ross' *On Death and Dying* [17]. Students are encouraged to refer to this and other literature in their written assignments as a way of linking theory, narrative, and medicine.

During their senior GP placement, their GP tutor identifies a suitable patient. A suitable patient for this teaching exercise is one who raises end of life care issues for doctors even if the patient and/or family may not raise these issues. These may include patients who have cancer, chest disease, dementia, heart failure, or stroke; they do not need to be in the terminal phase. Students then meet and talk with the patient on at least one occasion, usually in the patient’s home. Afterwards, students discuss the cases with GP tutors before writing the GP-based reflective essay. During their senior hospital medicine placement students are asked to independently identify a patient to interview from the patients they meet on the wards. Small-group case discussions are held with hospital consultants prior to writing the hospital-based reflective essay.

Students are asked to reflect honestly about their thoughts and feelings, emphasising that the confidential nature of their essays will be respected. A suggested structure outlines the main areas in which they will be assessed:

- Introduction to the patient, family and setting, including illnesses and treatments to date;
- Psychological, social and existential issues relevant to the patient and their family;
- Professional and ethical issues now or in the future, including future care planning, and how students would feel if they were the responsible doctor;
- Personal issues raised by the meeting and writing; now as a medical student, in the future as a doctor and as an individual;
- Learning arising from this clinical encounter and reflecting upon it.

Each section is marked on a scale of 0 (inadequate) to 3 (excellent), with a marking grid illustrating what satisfies each of these categories (Additional file 1). A zero in any category results in the essay being returned to student for further work. Additional marks are awarded for originality, personal insight and clarity. The assignment is a required course component: submission is a requirement in order to proceed to the final examinations, although not counting towards these examination marks. Formative written feedback is provided; to encourage students to engage with the assignment, the top ten assignments for each placement are awarded University prizes.

## Methods: researching the reflective exercise

During 2008 and 2009, two research projects investigated these reflective essays. This paper reports the themes identified in both projects regarding students’ views of the benefits of the activity. The Clinical School supported both studies. Protocols were submitted and approved by the University Psychology Research Ethics committee. For the first study, students were invited at a year-group lecture to give their signed consent for their essays to be included in the research study; 123 (86%) consented and an independent data manager anonymised essays (116 from GP setting and 118 from hospital setting). In practice, the majority of those who did not give their consent are likely to be students who were absent during the recruitment session, although this cannot be ascertained because of issues of anonymity. For the second study, students were given a presentation about the research prior to commencing this teaching element and asked to participate; all 146 students consented to take part in the self-reflection questionnaire and 107 (73%) filled in an additional evaluation questionnaire at the end of this teaching component. For each study, each student was assigned a random identification number (ID), which are included with the quotes to demonstrate the breadth of the dataset presented. For both studies written consent was provided for the data to be reported anonymously; consent was not obtained for the sharing of datasets or the essays in their entirety. Qualitative data for both studies was managed within NVivo.

The first project was an exploratory study. By drawing on theories about socialisation [18] and hidden and informal curriculum [19], it examined how students were writing about their encounters. It involved thematic discourse analysis, conducted by EB, SB, and SC, of 234 reflective essays from 2007 to 2008. What students wrote about the exercise itself and informal observation of several teaching sessions were included in the analysis. Previous publications have examined these essays with regards to medical professionalism [20] and students’ use the notion of denial [21]. Both of these papers discuss how, through these encounters and essays, the students were engaging with their professional identities and future role as doctors.

The second project evaluated this reflective exercise by assessing self-reflection conducted by RM. In 2009, a quantitative questionnaire instrument [22] was administered to all 146 final year students before and after writing their first reflective essays to see if the exercise changed their level of self-reflection, data from which is to be reported elsewhere [23]. To explore students’ perceptions of self-reflection and the reflective assignment, RM designed an evaluation questionnaire (Additional file 2), which 73% of students (sample size =107) completed anonymously. This paper presents a thematic analysis of students’ free-text comments from this evaluation questionnaire concerning their learning experience.

There was considerable overlap between the two qualitative datasets with regard to students’ interpretations of the experience, with particular convergence around the aspects students found to be valuable. For the purposes of this paper, we examined the qualitative data to review what the students said they found valuable (or not) from this palliative care teaching element, which involved patient contact and reflective writing.

Coding for ‘value’ was a subjective, interpretative endeavour. Coding relied on categorising text based on students’ use of language and assumptions about students’ responses to teaching methods. For example, a remark about writing the essay as being time-consuming was interpreted as a negative remark, even if a student did not explicitly state that they did not enjoy the activity because of this. Within the second project, collating free text with the other aspects of the questionnaire was used to corroborate coders’ interpretations.

Consequently, RM and EB discussed the two coding structures from each project, comparing the interpretive themes that each study had generated with this new research question in mind. The two coding structures from each project were combined using the groupings provided below, enabling triangulation of two qualitative data sources. The theme headings were derived from how the students labelled their learning experience. We have chosen to use these as they reflect what the students interpreted as valuable elements of the exercise. Coded sections were then re-checked by EB to ensure their fit within the new coding structure, including keyword searching for ‘value’ and ‘valuable’ of the original datasets, with some adjustments made. The purpose of this study was not to review if they were displaying reflective qualities in their writing, although educators on the course found having examples of this useful for future iterations of the teaching (see Discussion). The new analysis was presented to colleagues who teach on the palliative care course to elucidate their views and check appropriateness of the themes presented below.

## Results

When discussing the whole exercise of meeting patients, debriefing seminars and writing reflective essays, students described the experience as valuable, albeit challenging and time-consuming. Some of the most challenging aspects, such as having to sensitively talk to patients near the end of their lives, were also described as the most useful. Students reported that the exercise helped them identify their skills and what they hope to build on during their career. Overall, the great majority of students described the experience as valuable:

*“It was however one of my most valuable experiences as a student doctor, and I learned a great deal”. (Study 1:148-GP)*

### Dedicated time with patients

Students commented positively about the dedicated time they had to interact with and think about patients, which also included appreciating having time to interact with patients on a human level. They compared it to other aspects of their course where patients were the objects of study and the focus was on disease; in this exercise they were given the opportunity to know the patients as people and to consider the wider impact of illness. This often caused them to challenge their preconceptions about palliative care and interacting with dying patients:

*“I have learned that each terminally ill patient’s experiences and beliefs are unique to them and that palliative care patients cannot be ‘tarred with the same brush’” (Study 1:57-GP).*

In both settings, students commented on how it is a *“unique opportunity to build up a long term relationship with a palliative patient”* (Study 2:75). Within the community setting, students that were able to visits patients more than once particularly enjoyed this opportunity; this enabled them to ask follow-up questions, to see how things progress with time, and to build rapport with patients. For some, this was an opportunity to understand more fully what it could be like to be a practicing GP:

*“I am now aware of the large caseload of these cases within general practice and the importance of the GP’s role in patient care…” (Study 1:89-GP).*

Within the hospital setting, students were responsible for identifying a suitable patient, which enabled them to put their clinical training into practice. Again, having time to spend with patients was emphasised:

*“It was very useful to have a dedicated timeslot to have an in-depth conversation with a patient because as we move up the medical student ranks we tend to spend less time taking long, detailed histories and going into difficult issues” (Study 2:23).*

Students appreciated the time and patient exposure the interview element of the exercise provided them.

### Learning about the wider elements of treatment and holistic care

In some instances, students were surprised by how much the exercise taught them about the wider elements of treatment and care, learning about, for example:

*“The necessity of a team approach and co-ordination between team members, [and] the need to support the carer” (Study 1:85-GP).*

*“It helped me realise that asking patients about their home life is vital for planning their care and discharge from hospital…[and] that it may be important to consult other health care professionals in the hospital setting” (Study 2:28-HP).*

These were aspects of medicine that the students did not fully appreciate or articulate until they undertook this exercise.

Others found the exercise reminded them to see the patient in a more holistic view.

*“It is often easy to focus solely on the patient’s physical needs, such as pain relief, whilst ignoring psychological and existential issues, or simply provisions of home care packages and this is a lesson I will take forward into my foundation year practice and endeavour to apply as best I can” (Study 1:94-HP).*

Another student deconstructed this further, explaining that they learned that it is important:

*“to address the patient’s mood and feelings even if the questions seem hard to ask, because undoubtedly the answers will be harder to say but extremely important in the holistic care of the patient” (Study 1:30-GP).*

By being asked to consider the physical, social, psychological, existential elements, the holistic philosophy of palliative care was reinforced to the students. Through this they were able to understand how the different factors can impact a patient’s experience and care.

### Practice communication skills

Many of the students found the task of talking to seriously ill patients and possibly their relatives daunting. By the end of the exercise, they reported that it had been useful to practice some of the skills they had previously been taught:

*“I learned some valuable lessons about my style of communication in a situation such as this, especially giving space for the patient to talk as they wish to” (Study 1:62-HP).*

Students were able to identify specific aspects of their communication skills that they practiced.

The environment for this practicing of communication skills was important for some students, who stressed that the activity allowed them to implement the skills they had learnt in a real, but safe and supportive, environment:

*“It provided an opportunity to speak to a compliant palliative patient in a safe environment” (Study 2:64).*

In particular, this allowed students to go beyond their comfort zone when discussing difficult issues. One student was pleased that this allowed them:

*“to talk about existential issues without being awkward about bringing it up” (Study 2:47).*

Moreover, by asking them to reflect on the encounter, several students were able to identify what they would like to improve or how they would have done things differently. It was an opportunity to learn the interpersonal skills that role-playing in communication skills classes can only mimic:

*“This is a very important lesson for me to learn, that is to use my communication skills training to good effect and tackle such breaking bad news situations with care and consideration” (Study 1:61-GP).*

For some students, this was by far the most valuable element of the exercise and emphasised how the activity fit with other elements of their teaching.

### Learning about self through reflective writing

Some students displayed a level of self-reflection and were connecting the experience to their own personal development:

*“Through this reflective process you are able to identify exactly what you have learnt from an experience and to identify the gaping holes in your knowledge” (Study 1:82-GP).*

Through the reflective exercise students were able to comment on their own learning. This included learning the limits of their knowledge, confidence, and comfort:

*“I have also learnt a lot about myself; which topics I find easy to talk about, which I find difficult, and have started to think about why” (Study 1:99-GP).*

Many discussed how meeting patients and having to reflect on those meetings made them learn about how they cope and react to difficult situations:

*“It allowed me to reflect and better understand my approach to death and dying, and thus better prepared me to deal with similar situations I will undoubtedly face in the future” (Study 1:81-GP).*

Students therefore found it beneficial to reflect on their own limitations and to think about their professional development.

Because of these additional learning points, students found the exercise more rewarding than they expected. Although some reported that writing an essay meant more work and involved jumping through hoops, others found it a valuable way to demonstrate their learning to themselves:

*“Writing this reflective piece has also been helpful as it made me think about the issues involved on a deeper level that I otherwise would have done” (Study 1:76-GP).*

The reflective writing element of the exercise therefore allowed students to enhance the learning experience.

However, there were a number of students who disliked having to write about their experiences:

*“The encounter was valuable to me. I didn't appreciate having to write a long essay about it. I don't think it has made me a more reflective person” (Study 2:35).*

One student felt that having to formally reflect on their interactions with the patient *“worsened*” the experience (Study 2: 12). For some, the “*coursework seemed contrived*” (Study 2:13) and analysing the patient’s life felt *“artificial and unnecessarily intrusive”*, although they suggested that this may be a reflection on their unwillingness to analyse their own feelings (Study1: GP-23). These views were more prevalent in the free text element of the second research study than in the reflective essays themselves; four students wrote explicitly negatively about the writing element in the questionnaire compared to two within the essays. It is likely that students felt freer to express these views anonymously outside of the formally marked reflective essays, although these numbers are relatively small compared to the total sample size for each study. We have included these sentiments here as deviant case analysis, to acknowledge the range of responses to the activity, and to highlight what aspects some students did not find useful.

Overall, these research projects were able to illustrate how the students found the exercise valuable. For most students, not only was meeting the patient a useful experience, but reflecting on that encounter helped them to contextualise the learning:

*“I found the experience of meeting the patient very valuable, and it was useful to draw together what I had learnt in the essay afterwards. I think this will help the information and learning points to stick in my head” (Study 2:89).*

Students also appreciated the exercise, describing it as a *“privilege”* (Study 2:37) because of the exposure it gave them to different settings and people. The combination of listening to patients and reflecting on their discussions allowed them to view the *“human side of medicine”* (Study 2:100). In spite of complaints about the practicalities of the activity, students overwhelmingly reported that they were appreciative of the exercise because of the breadth and depth of the learning it provided.

## Discussion

Final year medical students found it valuable to meet patients in the palliative phase of illness, and to reflect on these encounters, at times in ways that they did not expect. While an emotionally and clinically challenging task, most reported obtaining benefit as they prepared for their future role as junior doctors, in keeping with previous studies [13, 24]. The teaching faculty are able to arrange two such encounters for all students (approximately 145-165) each year. Although the exercise was primarily designed to develop students’ knowledge, skills and attitudes in palliative care, the teaching team interpret the reflective element as adding value by encouraging students to think more broadly about patient care, their transition into becoming doctors, and how they related to dying and death.

### Palliative care teaching

Palliative care teaching has been increasing in medical schools within the UK and other parts of the world [5, 7, 25]. The findings from this study about what the students value about teaching in this area reflects findings from other research. For example, other studies have identified that students feel that teaching in palliative care helps them become and act as doctors, provides them with a holistic view, as well as providing stimulus to think and reflect, particularly about the personal and humanistic elements of care [26]. Recent research suggests that teaching in palliative care can improve students’ attitudes about the speciality [27]. Students in several studies have acknowledged valuing the practical and experiential elements of teaching [26, 28]. Similar to the findings here, when it comes to reflective practice, students may find the writing element time consuming [29] and difficult to express emotions, suggesting that alternative and creative methods also be explored in teaching although these may be more complicated to assess [30].

### Reflection and reflective practice

Those who review the essays annually observe that students each year are able to demonstrate the range of depth of reflection described by Niemi [31]. Generally, the most basic was *diffuse reporting* with unfocused description of the encounter. Others demonstrated *objective reporting* with only a descriptive account of what happened, but no evidence of reflection or how the experience affected them. Some demonstrated *emotional exploration*, giving evidence of the emotional impact of the experience with discussion about their own beliefs and values and how these have been challenged. Finally some achieved the greatest depth of reflective practice, *committed reflection* in which there was discussion of what had been learned, how it had affected them and how they feel that they have changed.

The activity described here is one way of incorporating reflective practice into medical education. As in other studies, it is not known whether the students were already naturally self-reflective and this was observed in their writing: they might have reflected on their experiences without the need for a formal reflective assignment [32]. Of course, there is some concern about the extent to which students are truthful in their essays about their experiences and reflections, as students seek to write to fit assessment expectations [33]. Quantitative data from the evaluation questionnaire, to be reported elsewhere, suggests that the reflective component of the assignment did increase the educational value of the clinical encounters [23]. Others have concluded that the use of reflective writing assignments in undergraduate education needs to be part of a wider programme of teaching reflective practice that includes one-to-one mentoring and reflection in small groups [34, 35].

### Development and improvement of the teaching

The findings of these studies have highlighted the aspects of the exercise that students found valuable: the consolidation of communication skills training in potentially challenging conversations, combined with the holistic approach of palliative care, was valued by many. The objectives of the teaching element have subsequently been expanded to address these new learning points. An expanded initial introductory lecture now includes information on the four levels of reflective practice outlined above [31] with anonymised quotations from previous students reflective essays illustrating each level. Teaching what critical reflection looks like improves student performance in these kinds of assessments [36]. The central role of reflective practice in their future careers and annual appraisals is emphasised, as is the value of meeting patients and their family members on more than one occasion.

The findings have also been used to evaluate this course component, which has enabled the optimising of learning through insights not obtained from standard feedback surveys. When first introduced in 2007, the objectives of this teaching component were initially modest: now several years on, these have revised in light of the progress made and the research conducted, including asking students to think critically about any ‘learning arising’ from their encounters and reflections. As a result of these studies, students are now taught about what different levels of reflection and what they look like. Additionally, one future development will be moving the General Practice case to the fifth (penultimate) year, in a new longitudinal format in which students will revisit patients on several occasions throughout the year as students valued having this experience and ability to build up rapport with a patient.

### Limitations of the study

As with any student evaluation, there are questions about the validity of the feedback to capture and assess the quality of the teaching [33]. We have chosen not to rely solely on the text provided within the essays themselves, although this is the larger dataset, because we acknowledge that students may be inclined to write positively about the exercise in order to demonstrate reflective practice. Although not all students did this and some wrote negatively about the activity, as described above; however, more students wrote negatively about the activity in the second project where their comments were explicitly part of an anonymous course evaluation. However, due to the methods chosen for the individual projects, it is not possible to weight these comments across the study. Therefore, we are only able to report on the themes and variety within them, instead of ranking elements of the teaching experience in terms of value from the students’ perspective. This study represents what students valued about the particular teaching element at the time of writing their essays/evaluations; it cannot provide us information on what values they attribute to the teaching as they progress in their careers. Lastly, this paper is reporting the teaching and evaluation of one medical school that has high entry requirements; what these students express as being valuable may not be representative of wider student populations.

## Conclusion

The purpose of this paper was to describe and examine what students found valuable about the teaching component involving palliative care encounters and reflective essay writing at the University of Cambridge School of Clinical Medicine. It is possible to arrange for all medical students to individually meet and talk to patients who are receiving palliative care, in both general practice and hospital settings. Requiring students to write reflective essays about these experiences is one way to incorporate reflective practice into these encounters and to assess the learning gained from them. Most students described both the meetings with patients and the essays as valuable elements of their education, providing many learning points, including experiential learning about holistic care and communication skills. As to be expected, a minority of students reported to not value having to write reflective essays. The combined results of different studies that investigated the encounters and reflective practice from different perspectives was useful in identifying the key elements of the added value of this component of the Cambridge course.

## Additional files

Additional file 1: Assessment grid for reflective essays. (DOCX 62 kb)
Additional file 2: Evaluation questionnaire. (DOCX 57 kb)
