# Supplementary material for: Learning to care: medical students’ reported value and evaluation of palliative care teaching involving meeting patients and reflective writing
Source: BMC Med Educ. 2016 Nov 25;16:306. doi: 10.1186/s12909-016-0827-6 (PMC5124265; doi:10.1186/s12909-016-0827-6)
Supplement: Additional file 1: — Assessment grid for reflective essays. (DOCX 62 kb) [file 12909_2016_827_MOESM1_ESM.docx]

Additional File 1: Assessment Grid

The following grid is used for assessment and feedback (maximum 17):

|  | **0 = inadequate** | **1 = adequate** | **2 = good** | **3 = excellent** |
| --- | --- | --- | --- | --- |
|  | Inadequate information / diffuse reporting | Basic facts / Objective reporting | Good information / Emotional exploration | Succinct information / committed reflection |
| **Introduction & background** |  |  |  |  |
| **Psychological, existential and spiritual issues** |  |  |  |  |
| **Professional:**  **past, present and future care and ethical/legal issues** |  |  |  |  |
| **Personal issues: as medical student, as future doctor and as an individual** |  |  |  |  |
| **Learning arising: from this patient and for future patients** |  |  |  |  |

| **Additional Marks** | **Range 0 to 2:**   - **0 to 2** for **Professionalism** - **0 to 1** for **Referencing** |
| --- | --- |
