# Supplementary material for: Learning to care: medical students’ reported value and evaluation of palliative care teaching involving meeting patients and reflective writing
Source: BMC Med Educ. 2016 Nov 25;16:306. doi: 10.1186/s12909-016-0827-6 (PMC5124265; doi:10.1186/s12909-016-0827-6)
Supplement: Additional file 2: — Evaluation questionnaire. (DOCX 57 kb) [file 12909_2016_827_MOESM2_ESM.docx]

Additional File 2: Evaluation Questionnaire

| *Please rate your agreement with the following statements on the scale provided and answer the questions below:*   1. **For me this educational experience was:**  \| Very valuable \| Valuable \| Neutral \| Of little value \| Of no value \| \| --- \| --- \| --- \| --- \| --- \|  1. **Why was that?** 2. **What could be improved?** 3. **For me, writing the reflective essay after meeting the patient:**  \| Improved the learning experience greatly \| Improved the learning experience a little \| Made little difference to the learning experience \| Worsened the learning experience a little \| Worsened the learning experience a lot \| \| --- \| --- \| --- \| --- \| --- \|  1. **Why was that?** 2. **Has your experience of self-reflection changed as a result of this assignment?** |
| --- | --- | --- | --- | --- | --- | --- | --- | --- | --- | --- |
